# Carcinogenesis and Chemotherapy Viewed from the Perspective of Stoichiometric Network Analysis (SNA): What Can the Biological System of the Elements Contribute to an Understanding of Tumour Induction by Elemental Chemical Noxae (e.g., Ni, Cd) and to an Understanding of Chemotherapy?

**DOI:** 10.1100/tsw.2003.01

**Published:** 2003-05-05

**Authors:** Stefan Franzle, Bernd Markert

**Affiliations:** Department of Environmental High Technology, International Graduate School (IHI) Zittau, Markt 23, D-02763 Zittau, Germany

**Keywords:** cancer, metals, inhibition, cell cycle, stoichiometric network analysis, phase shift, inhibition

## Abstract

The biological application of stoichiometric network analysis (SNA) permits an understanding of tumour induction, carcinogenesis, and chemotherapy. Starting from the Biological System of the Elements, which provides a comprehensive treatment of the functions and distributions of chemical (trace) elements in biology, an attempt is made to interrelate the essential feature of biology and — regrettably — of tumour genesis by superimposing SNA reasoning on common features of all crucial biological processes. For this purpose, aspects, effects and drawbacks of autocatalysis (identical reproduction which can occur either under control or without control [in tumours]) are linked with the known facts about element distributions in living beings and about interference of metals with tumours (in terms of both chemotherapy and carcinogenesis). The essential role of autocatalysis in biology and the drawbacks of either controlled or spontaneous cell division can be used to understand crucial aspects of carcinogenesis and chemotherapy because SNA describes and predicts effects of autocatalysis, including phase effects that may be due to some kind of intervention. The SNA-based classifications of autocatalytic networks in cell biology are outlined here to identify new approaches to chemotherapy.

### INTRODUCTION

This study is concerned solely with *chemically induced* carcinogenesis and with chemotherapy, in particular with processes in which metal ions attack nucleic acids directly. The irritant and inductive effect of inhaled particles 1–4 µm in size on the genesis of lung and bronchial carcinomas (such as asbestos and glass fibres) and the carcinogenic activity of arene epoxides (formed from benzene, benzo(a)pyrene, cholanthrene etc.)[1] will not be considered. Likewise, direct or indirect (boron-10-neutron capture[1] based[2, 3] radiotherapies are beyond the scope. The link between chemical reactions of ambient chemicals and deletions of some parts of a genome — which might bring about cancer — is the environment (besides the interior of some cell or organ). There will not be any carcinogenic effect unless the compound is taken up from around. Moreover, redox speciation (often depending on $MnO_2$ concretions), biomethylation, and epoxidation by either organisms or by atmospheric compounds such as ozone or nitrogen dioxide can thoroughly alter bioavailabilities and carcinogenic potentials of both organic and inorganic materials. This holds true for all chromium, lead, arsenic or PAHs. Additionally there are similarities between features controlling geochemical distributions — e.g., via solubilities of phosphates — and certain ways of attack on nucleic acids (f.i. for Be and As).

The corresponding biogeochemical changes and cycles can likewise be described by means of Stoichiometric Network Analysis. Chemical features pertinent to speciation thus influence geobiochemical distribution patterns and are likely to exert some drawback on the cell cycle. Hence both actual expositions and carcinogenic risks by environmental "pollutants" can be evaluated from a nested approach. Biogeochemical cycles with respect to ecosystem stability were discussed before[4].

It was and is commonplace to evaluate possibly carcinogenic materials released to the environment with empirical information but identifying possible causes of cancer in cell cycles derived from systems theory should allow us to *predict* cancer hazards by pieces of information from both general inorganic biochemistry and the Biological System of Elements. Given this, a comprehensive treatment of "environmental cytology" can be established.

However, stoichiometric network analysis also permits for an understanding of interactions of metal chemotherapeutics with metalloproteins that inhibit enzyme activities rather than directly attack nucleic acids, because the latter enzyme processes are in a certain sense also autocatalytic[2] within the larger framework of a cell[3].

A practical example of this would be the blocking of Fe-containing enzymes by gallium(III) salts in chemotherapy[5,6].

The starting point for this study is again the Biological System of the Elements[7,8,9], that covers, in its currently available form, most of the elements discussed here and their abundance

[1] $^{10}B + n \rightarrow {}^{7}Li + \alpha$; both α-particles and $^{7}Li$ causing radiation damage in the close vicinity of the body region where boron was present (tailor-made compounds like boron clusters such as $[B_{12}H_{11}(SH)]^{2-}$ are used that undergo enrichment in tumours, with or without isotopic enrichment in boron-10 [the natural contribution being 19.6% of isotopic mixture]).

[2] Autocatalysis means self-related reproductive catalysis with the potential for constant acceleration of the reaction until at least one resource is exhausted. Catalysts always promote forward and reverse reactions to the same extent, with the result that they do not change the position of the kinetically definable chemical equilibrium. This means that the passage through an autocatalytic loop and its — possibly non-linear — reaction to changed ambient conditions is usually accelerated.

[3] Although both metalloproteins and nucleic acids — besides a lot of other compounds — are essential for proper functioning of a cell or whole organism and are reproduced upon budding of cells or reproduction of the entire organism in an autocatalytic fashion, neither nucleic acids nor metalloproteins do replicate autocatalytically *sensu strictu* in vivo, that is, without an interference by some co-catalyst. Hence there is no theoretical reason to employ a different theoretical approach towards autocatalysis in RNA or DNA as opposed to metalloproteins; rather, SNA can be used to understand both kinds of processes including their possible perturbations. The common precondition here is the general condition for sustained life yet also applies to tumours because there is no beforehand assumption on control of autocatalysis. The latter can be inferred on both the activity of certain biogenic or xenobiotic compounds including carcinogenic and cytostatic compounds. For un-perturbed AC cycles, the result apparently is universal.

correlations. It originally established a collective abundance correlation between different chemical elements in a specific group of living organisms, namely vascular plants.

Starting from statements with such a fairly limited scope, the present authors used stoichiometric network analysis (SNA; for details see below) to develop a more general method of describing vital processes on the basis of their autocatalytic aspects. In the present study this method is extended to cover processes that are simpler than the usual ones in multicellular organisms (including vascular plants) in that they are not subject to regulation. And that is exactly what pathologically uncontrolled cell division in tumours is.

If vital processes are considered in terms of SNA[9], it is also possible to draw formal conclusions about the mode of action of cytostatic agents.

This was first demonstrated for the inhibition of metalloenzyme activities by gallium salts; it has since been possible to extend the method of description to the nucleic acid replication cycle. This cycle is the point where most chemotherapeutic agents that rely upon metal complexes attack. The relationships and any dynamics that may result (occurrence of spontaneous, uncontrolled oscillations in the intracellular autocatalytic cycle as a possible cause of chemically induced tumours) are discussed formally with the aid of SNA. The focus of this study is on metal ions[4] and some of their complexes and compounds in their role as both carcinogens[5] and cytostatic drugs.

### GENESIS OF TUMOURS: THE THREE-PHASE MODEL

Tumour formation begins with individual cells or small numbers of cells that have withdrawn from the body's or the organ's own system of regulating cell division after damage by chemicals, ionising radiation or the like. The next step is a failure of the immune system. The "budding" tumour cell only becomes pathogenic if and when it is able to avoid cell communication and subsequent apoptosis "orders." Proliferation into tissue differentiated in a different manner (metastasis) raises additional theoretical problems in respect of cell communication.

In the course of tumour formation, it is possible to distinguish between different phases and degrees of "efficiency" in factors that give rise to tumours chemically[6] Boutwell et. al [12] distinguish between normal cells, initiated but dormant tumour cells, and finally those of a

[4]Whereas in a comparison of the different species of an element the highest values (i.e., the smallest $LD_{50}$ quantities, most pronounced relative toxicities) for acute toxicity are generally found for the aqua ions (apart from alkylated derivatives of the notorious tributyltin or alkyl/aryl mercury cations) or their protolysis products, i.e., in some cases bridged hydroxo species (Al, Cu(II), Pu(IV)), the sequence for carcinogenicity — for example that of nickel — is totally different and not capable of generalization in the above manner[10]. Oxoanions such as those of arsenic, vanadium, or chromium can easily enter the cell nucleus with the transport mechanism for sulphate and be "confused" with phosphate by transport proteins. After incorrect incorporation this may result in breaking of the polyphosphate chains both in nucleic acids [which may be repaired] and in energy metabolism [nucleoside triphosphates]. After reduction to As(III) or V(IV) they also intervene in other biochemical processes; As(III), for example, is bound very strongly to the sulphur sites of thiolates (e.g., cysteine).

[5]German law on chemicals already has no levels that may be regarded as harmless for carcinogenic substances such as benzene; this is in contrast to "merely" toxic substances (like hydrogen cyanide) for which MAK (maximum workplace concentration) and MIK (atmospheric concentration close to the ground) values are defined in the context of occupational medicine. But here there is a prospect of identifying particularly dangerous carcinogens theoretically in advance on the strength of their mode of action. Species such as chromium (as chromate (VI)), which are capable of altering the frequency of the autocatalytic cycle, must be regarded as especially hazardous.

Carcinogens of this kind may act on autocatalytic 1B or 1C loops (see below) in different ways. The function of Z as an added or lost species in the autocatalytic cycle distinguishes the types 1B and 1C from each other. Conversely it is possible to conclude that substances with a cytostatic effect inhibit the corresponding cyclic processes by transforming strong, marginally strong or critical cycles into weak ones. An example of this is the unspecific cytostatic effect of Ti(IV) complexes[11], which inhibit proteases, probably by attaching themselves to serine residues.

[6]The rather complicated way of expressing this stems from the fact that the Boutwell model includes not only oncogenes such as benzo(a)pyrene but also promoters and further substances. It also considers the manner in which these interact.

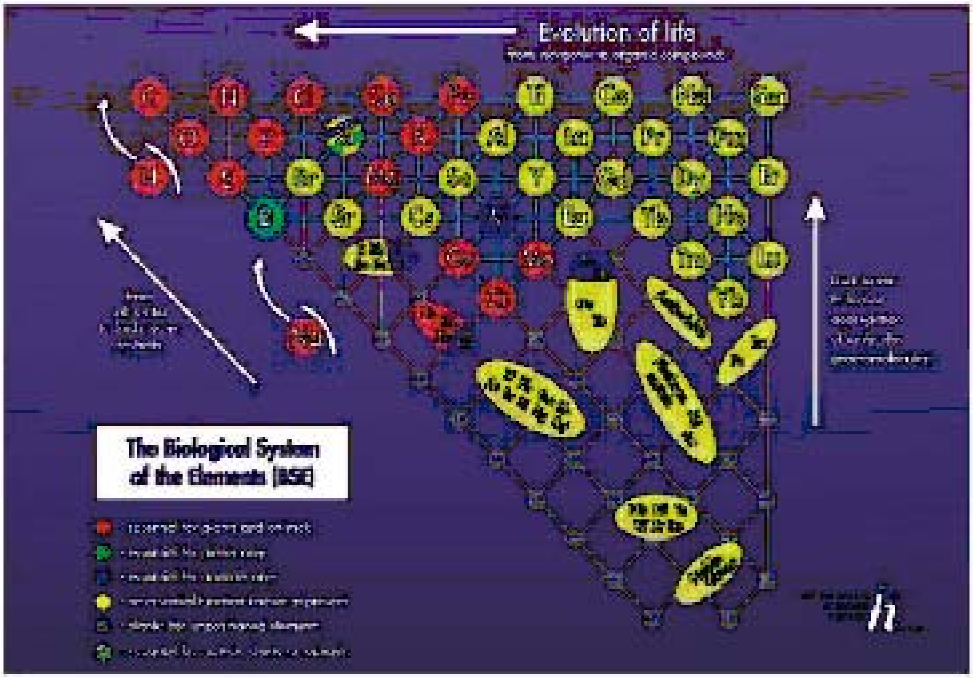

**FIGURE 1.** The Biological System of the Elements[7].

growing tumour. An initiated cell still has the phenotype (microscopic appearance) of a normal cell but the biochemical potential of a tumour cell; rapid cell division is, however, suppressed. Initiation or incomplete carcinogenesis is caused by limited doses of a carcinogen followed — in the presence of a promoter or continued application of the carcinogen — by promotion of the pre-neoplasm and conversion of its cells. Further application of either the carcinogen or the promoter results in propagation of both dormant tumour cells and those of tumours that are already growing. In these phases the tumour has overcome the repressive influence of the immune system on the dividing cells; this allows it to continue growing and possibly to metastasise.

### CARCINOGENS AND CYTOSTATIC AGENTS: MODES OF ACTION THAT SOMETIMES CO-EXIST IN THE SAME SUBSTANCE

Numerous carcinogens act by way of process cascades involving reactive forms of oxygen, including superoxide/$HO_2$ and the OH radical. Among them are some in which this is not obvious, e.g., numerous nickel compounds. Conversely, superoxide dismutase containing Cu and Zn prevents chromosome breakage even under the influence of phorbol esters.

The same chemical species may have both a cytostatic and a carcinogenic effect (for instance, $As_2O_3$); however, this may simply be due to the fact that the effects differ in different tissues, so they do not influence the same (here undesirable) enzyme functions. A model that describes the real biochemical activity by means of stoichiometric network analysis and studies and compares the coupling of the metabolic processes of different cells and tissues with regard to their propensity for interference leads us to expect this.

Unlike the majority of organic chemical reactions, most biochemical reactions are *reversible* (e.g., transamination; esterification; carbon-carbon cross-linking to, with, and through carbonyl functions; redox processes with quinones or flavines, the entire tricarboxylic [citric] acid(-s) cycle); this appears to have a biochemical advantage. Even reversible[7] reaction systems may result in chemical oscillations[8], e.g., the iodine/iodate/arsenic(III) system. But unlike most biochemical processes, the transfer of information from the nucleic acid to a protein takes place in *one direction only.*

If the sequence of the latter is altered, for example, if parts of the protein chain are cut off and put together differently, the result is not a change in the genome or even in individual nucleic acids (i.e., there is no Lamarck-type behaviour on the molecular level — the central dogma of molecular biology). Here the remaining metabolic processes differ fundamentally in respect of the preservation of information from those affecting the genome; for this reason the discussion that follows is restricted to substances with a genotoxic or cytostatic effect that interact with nucleic acids directly.

### LIST OF THE METAL-BASED CHEMOTHERAPEUTIC AGENTS

We now have a very good understanding of the roles of certain chemical elements (Ti, Fe, Cu, Pt) in the treatment of tumours[11], so the relevant biochemical processes and consequences can be investigated by formal methods such as those of stoichiometric network analysis.

The following table shows the metal-containing chemotherapeutic agents already established or tested in preclinical trials/animal experiments or on cell cultures; they are arranged according to groups, ligands, and sites of action or types of tumours, which are sensitive to the agent. The ligands are especially important for absorption, but experience with metal salts shows that they are also significant for acute toxicity and thus tolerance/side effects of the drugs (summarized from Guo and Sadler[6] and other sources):

So most of the metal compounds recognized as effective against (some) tumours react directly with nucleic acids and add on to these. In some cases the exact binding sites and the topological effects (with respect to helicity) of such binding on the DNA are not known; in other cases (including cis platinum) they depend on the concentration or constitute equilibria of different forms.

The task of functional classification of chemotherapeutic agents was addressed before[13], but not with regard to the most common and essential feature of biology that exactly cause the problems with tumours if they are left or become uncontrolled, that is, autocatalyis or biochemical replication. The formal method chosen here to investigate the possible dynamics of the system is called Stoichiometric Network Analysis[14,15,16]. An SNA analysis of the effect of therapeutic agents can and must start from the replication of both normal and metal-complex-modified nucleic acids as a special instance of autocatalytic behaviour. Besides the positive nucleic acid strand, both negative matrices and the replication of catalysing proteins (polymerases) are involved in this process. The autocatalytic cyclic processes of the nucleic acids are linked to the activities of polymerases and other components involved in replication. But the

7 Both the oxidation of organic compounds such as malonic acid, sulphite, or thiocyanate and the reduction of chlorite or chlorine dioxide are irreversible under the conditions prevailing here.

8 Besides suitable reaction kinetics, concentrations of the reactants, and pH values, the condition for the occurrence of oscillations is the coincidence of autocatalysis (reproduction of a catalyst during the reaction, e.g., release of protons in oxidation with an acid catalyst) with feedback inhibition, i.e., the introduction of a side reaction that has an antagonistic effect in that it uses up part of the autocatalyst. The latter prevents both the explosive course of the reaction and a mere time reaction with the successive conversion of two substrates; the conversion of the two substrates (e.g., of iodine and malonic acid in $ClO_2$-based oscillators or of hydroxylamine and sulphite into Landolt clock reactions with iodate) cannot then take place selectively in sequence if the conditions for continuous autocatalytic acceleration of one of the reaction paths are disrupted by an antagonist.

latter are also produced according to a genetic programme, which means that they are dependent on the nucleic acids in whose replication they are involved. These overall conditions are the

**TABLE 1**
**Chemotherapeutic Agents, Their Biochemical Sites of Action and Lines of Tumour Cells Against Which They Are Effective (In Vitro)**

| Metals[9] | Ligands | Tumours | Sites of Action |
|---|---|---|---|
| **Main Groups** | | | |
| Ga(III) | Water[10], maltolate, oxine[11] | Lung, bones | Fe-antagonist |
| Ge(IV) | Alkyl[12] | Various[13] | RNA + DNA synthesis |
| As(III) | Oxide (in form of $As_2O_3$)[14] | Promyelot. leukaemia | Causes apoptosis |
| Sn(IV) | Alkyl + peptide[15] | **p 388** Leukaemia[16] | Unknown, not DNA |
| **Transition Groups** | | | |
| Platinum Metals | | | |
| Ru | Chloride, N or S donors[17] | Lymphosarcoma | Transferrin receptor |
| Pt(II) | *cis*-di-amines + anions[18] | Liver, bladder | DNA (insertion) |
| Pt(IV)[19] | like Pt(II) complexes + iodide[20] | Bladder | DNA (insertion) |
| Rh(I)[21] | 1,5-Cyclooctadiene + diverse[22] | Primary; lung metastasis | Purine bases |
| Subgroups 4–7 | | | |
| Ti(IV) | 3-ketoenolates, alcoholate[23] | | |
| Ti(IV) | cyclopentadienide[24]+ $Cl^-$ | Breast, digestive tract | DNA (phosphate chain) |

[9]Arsenic and germanium are non-metals or “semimetals” at most (no aggregation); rhenium is a metal but with a very pronounced covalent chemistry. This is also true of tin.

[10] he usual pharmaceutical form is gallium(III)-nitrate-hydrate.

[11]Oxine = 8-hydroxyquinoline, a virtually universal chelate ligand for “hard” metal ions.

[12]“Spirogermanium” = 2-aza-8-germaspiro[4.5]decane-2-propanamine, 8, 8-diethyl-N,N-dimethyl-,dihydrochloride, a germanium-organic compound thought to be highly stable to redox reactions on Ge.

[13]Leukaemia; bladder, breast, ovarian, rectal and other tumours, sarcomas. But the substance is highly neurotoxic and can trigger symptoms of epileptic seizures.

[14]Approved by the FDA in 1998 under the name Trisenox® for treating forms of leukaemia resistant to therapy.

[15]N-butyl (bound directly to tin) + glycylglycine (dipeptide); it is a dialkyltin compound (less toxic than trialkyls, more effective than monoalkyltin derivatives).

[16]The substance has no effect on many other tumour cell lines.

[17]Dimethylsulphoxide (= DMSO, bound to S), imidazole, synthetic multidentate amino acids such as propylene-diamine tetraacetic acid; the monodentate ligands (DMSO, imidazole, chloride) are instable to hydrolysis (which results in activation for addition to the transferrin receptor and interference with iron metabolism etc.).

[18]Chloride, hydroxide, multidentate carboxylates (oxalate, lactate, glycolate, cyclobutane-1,1-dicarboxylate [Spiroplatin®] etc., but no amino acids).

[19]Can be activated either reductively (thermal reaction of platinum(IV) with glutathione or other thiols) or photochemically (photoreduction by visible light with expulsion of iodine).

[20]Further Pt(IV) complexes for oral administration that are probably reduced thermally or enzymatically in vivo before a reaction with DNA takes place are *trans*-$[Pt(OH)_2{}^{cis}(isopropylamine)_2Cl_2]$ (Iproplatin®) and $[PtCl_4$(1,2-diaminocyclohexane)] (Tetraplatin®).

[21]Still at the trial stage. The complexes investigated to date are easily oxidized biochemically to form scarcely active or inactive octahedral Rh(III) species that may therefore intercalate less easily into the DNA, although Rh(I) is a very stable oxidation state. Like their cobalt and iridium counterparts, the Rh(III) complexes are also highly inert to a ligand exchange that is not accompanied by redox reactions. The dialkene ligand seems to remain bound to the rhodium and does not interfere with addition or intercalation.

[22] Chloride, $NH_3$ or amines, 3-ketoenolates etc.

[23]Budotitan®: enolate anion of benzoylacetone, combined with ethanolate (each ligand occurs twice in the complex), Ti(IV).

[24]Cyclic aromatic carbon anion ($C_5H_5^-$, Cp for short). Those derivatives with functionalisations (side chains, even methyl groups) at the cyclopentadienide ring do not have an anti-tumour effect[6], whereas ligands other then Cp can be varied. All the complexes mentioned here are metallocenes (bis-cyclopentadienidometal sandwich complexes) with additional ligands unstable to hydrolysis; V(V), for example, reads as $[Cp_2VCl_2]^+$. The cationic nature and the chloroligands are more significant for solubility in water and thus the pharmacokinetics/administration paths of the complexes than for reactivity to nucleic acids.

| | | |
|---|---|---|
| V, Mo, Re(V) | cyclopentadienide + $Cl^-$ | Proteins acting on DNA[25] |

boundary conditions of an SNA analysis (in the context in which apoptosis is promoted by the chemotherapeutic agent, etc.).

### STOICHIOMETRIC NETWORK ANALYSIS (SNA) AS A STRATEGY: POSTULATES AND CONSTRAINTS FOR ITS APPLICATION TO BIOLOGICAL SYSTEMS AND QUESTIONS

The term "autocatalysis" describes a certain type of cyclic processes in which several times the original amount of the catalytically effective substance is produced by (usually) chemical processes. This is a fundamental phenomenon of biology in general — not only of nucleic acids — and can be described by relatively simple formal means. Living systems behave catalytically through their (ability to) reproduce if they contribute chemically to their own reproduction. Our aim is to understand the "role" played by a substance (the term "function" is scarcely appropriate for a carcinogenic agent) and ideally to identify the types of autocatalytic cycles in which it is involved. The classification would then give useful information for a chemotherapeutic "attack" or preventive measures for persons exposed to carcinogens.

Stoichiometric network analysis (SNA) takes into account two important approaches toward an understanding of dynamic and topological features of structures or systems which, considered in their complete forms, would be intractably complex[26]:

1) A systematic simplification of the autocatalytic system under review is achieved without any losses in depiction of the dynamics, clearly emphasizing the causes of autocatalytic behaviour, the nature of the factors or substances controlling it, and their sites of action;
2) A diversity of mathematically substantiated theorems (cf. [14,15]) that can be used directly to describe biological systems. This minimizes expensive and time-consuming computer simulations that are often difficult to apply; qualitative statements (both ecological and biochemical) based on SNA have proved reliable many times on various hierarchical levels[9,18].

According to SNA it generally holds true that the ability of any autocatalytic cycle to function (in this case the organism's ability to survive or the aggressiveness of the tumour) depends on how the formation and reproduction of the autocatalyst relate quantitatively and mechanistically or topologically (in respect to the structure of the reaction network) to those processes that inactivate the autocatalyst (antagonists), break it down, destroy it, or remove it from the system (excretion via metabolism, formation of volatile forms and such that pass through the kidneys). We are concerned here with an understanding of "exotic"[27] behaviour of chemical systems[19] besides that of biologically or medically relevant processes.

The result is a system of two linked loops; the first of these contains the autocatalyst X, while the two cooperate with and partially control each other. Their behaviour can already be analyzed formally with qualitative arguments; the loop determining the flow of nucleic acid and also comprising polymerases being regarded as the outer one. An outer loop of this kind occurs in

[25] Topoisomerase II, protein kinases etc.; the analogous niobium complex does not react with nucleic acids.

[26] SNA has already been used to achieve a semiquantitative understanding of the role of biogeochemical cycles in global climate, regulation of marine salt content, the "strategy" employed by pathogenic viruses such as HIV and pattern formation processes in chemical reaction-diffusion systems[17].

[27] These include oscillations and bistability, which are also found in biochemical systems.

all critical[28] (see below) types of autocatalytic cycles (Type 1)[20] that are extremely heterogeneous both structurally and dynamically; however, its functions differ considerably.

### APPLICATION OF SNA TO THE "BIOLOGY OF METAL(OID)S"

In SNA, both essential and toxic elements are classified by means of their dose-effect curves according to how the autocatalytic effect changes with concentration[18,21]. This method is easily applicable even if there is no threshold value in the sense of a No Observed Effect Level — as in the case of carcinogenic substances — and even if elements that are assumed (V, As) or known (Co, Ni) to be essential to man are carcinogenic or toxic to reproduction (Mo, Zn) in an overdose and/or in a "biochemically useless" speciation form. The reason is that the dependence of autocatalytic activity on concentration is the crucial factor here, and abnormal (absent or irregular) cell division is regarded as a disruption/alteration of precisely this autocatalytic activity.

Qualitative investigations by SNA therefore seek to draw a comparison between the reaction *orders* of autocatalysis and those of loss processes; they are sufficient and suitable for our purposes. The "roles" of substances are determined in the context of the classification of autocatalytic systems; it is possible to show that one of the elementary, completely reduced[29] "skeleton mechanisms," namely 1B[20,22], adequately reflects most of the processes relevant here and can therefore serve as a starting point for further measures, i.e., the design of new chemotherapeutic agents on a chemical process and dynamics centered basis.

### CLASSIFICATION OF CYCLES IN STOICHIOMETRIC NETWORK ANALYSIS

In general, SNA distinguishes between strong, critical, and weak (autocatalytic) cycles, hetero- or cross-catalytic cycles, and finally marginally strong cycles. The following applies to such cycles.

The qualitative statements relevant to our problem include the following (thus defining boundary conditions):

[28]The existence of an outer, cooperative cycle makes both of the connected cycles critical, a necessary condition for the long-term coexistence of the systems (c.f. Clarke's treatment of Eigen's hypercycles and other systems of this kind [15]). To disrupt either the activity of the polymerase or the reading of the nucleic acids is the main objective of metal-based chemotherapy (see table); at the same time, interactions (rarely used therapeutically) occur with other metal ions and their biochemical functions or (metal [here Fe(III)] dependent enzymatic) autocatalytic cycles (e.g., gallium).

[29] In this case *elementary* means that only one autocatalyst exists (though it may pass through several forms or intermediates before being regenerated, constructing but a single loop) and regulates the behaviour of the system; *reduced* describes the abstraction of the network down to the smallest skeletal structure that still reflects all the dynamic properties. SNA reliably permits such an extent of reduction. The components of the skeletal mechanism can be added pictorially and topologically in a modular manner[20], allowing to add certain such modules to a given skeleton mechanism purposely to induce novel dynamic features. In this way it is possible, in practice, to transform an autocatalytic, acid-catalyzed clock reaction into an oscillator or pattern-forming system by making certain functional additions, i.e., adding certain compounds.

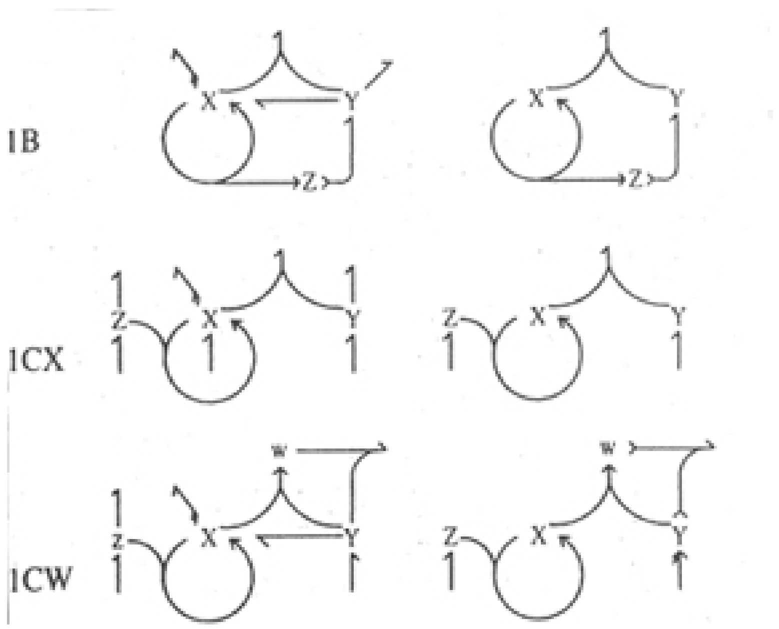

**FIGURE 2.** Diagrams of the Cycles 1B, 1CX, and 1CW (after[20]).

### TABLE 2

### Basic Types of (Auto)catalytic Cyclic Processes a) Autocatalytic process order > exit order
b) Autocatalytic process order = exit order
c) Autocatalytic process order < exit order
d) No autocatalysis, but heterocatalysis (applies to numerous geochemical background processes, e.g., [23][30], and
e) **Cross** catalysis, including symbioses a) corresponds to a strong (current) cycle,
b) to a critical cycle, and
c) to a weak cycle;
d) is also a weak cycle, but it cannot be dominated biologically since reproduction no longer plays any substantial role in a reproduction order of $O_{ak} \approx 0$;
e) is a critical cycle by definition because of mutual dependence — and has unstable long-term kinetics[31]. The nature of the cycle may change through cross-connection of reaction loops among themselves, the addition of highly reactive antagonists, the polymerization of the autocatalyst and the like, but always in the direction of weaker autocatalysis[15].

30 The redox reaction between $NO_3^-$ and Fe(II), that produces ammonium ions but inhibits both nitrate respiration and the activity of iron bacteria, is not catalyzed by their iron-containing product magnetite; it is, however, catalyzed by Cu(II) and goethite, which is not formed under the geochemical conditions[23]. In this case heterocatalysis takes place *instead of* (biotic or inorganic) autocatalysis.

31 Similarly, symbiotic systems such as lichens are particularly suitable for identifying interference such as the influence of environmental noxae[21].

Every natural autocatalytic process contains (at least) one (material) feedback loop, and the exchange of materials may also be regarded as an exchange of information[32]. In multicellular organisms the reproductive processes on different levels of the system include

- reproduction of an entire organism (sexual or otherwise, with various autocatalytic orders, sometimes even alternating)
- cell division
- reproduction of certain (in fact, almost all) proteins — e.g., of such containing metals nucleic acids

SNA is appropriate for each of these levels, which are at least three in number and mutually interdependent because autocatalysis is the crucial feature to be investigated in terms of control and dynamic stabilities.

Being a formally autocatalytic process, cell division implies efficient autocatalytic cycles that are capable of oscillation and tend to be unstable in other respects, too. This means that they require regulation to the point of apoptosis (induced and controlled cell death) if multicellular life is to be possible[33]. Each kind of differentiated tissue, i.e., every type that acquires its physiological function in a multicellular organism (embryonic growth) or retains it and is not (or is not yet) a tumour is characterized by a particular set of currently expressed[34] genes and thus realized biochemical functions, each of which may be associated with an autocatalytic function of a metal ion in a metalloprotein.[35]

[32] In accordance with

$$n*X + k*S \rightarrow (n + 1)*X + P$$ (the autocatalytic *order* being [n + 1]), autocatalysis is bound up with the release of *additional* autocatalyst X and usually further products P (for example, those of reduction products, in addition to protons, in reactions with an acid autocatalyst [clock reactions]; this means that the reaction, which tends to become steadily faster in the case of (strong) autocatalysis, alters the environment and thus *informs* other entities in the adjacent area accessible to diffusion of what is taking place (since intercellular communication mostly takes place chemically, not via electric impulses as is the case with nerve-dependent reactions). The information theory is therefore fully applicable to autocatalytic systems[24].

Moreover, such (strongly or critically) autocatalytic systems are *excitable*, so that chemical waves (trigger waves)[25] run through the medium and speed up the spread of information. In biology (embryonic growth) these are chemical waves of $Ca^{2+}$ ions on the cell walls. There are far more wave-forming autocatalytic systems than systems that behave as oscillators.

[33] It is a noticeable fact that the first multicellular organisms did not appear until about 1.4 billion years ago, i.e., after more than 60% of the entire period of biological evolution up to the present had passed and without any recognizable connection with drastic geochemical or climatic changes (the "Precambrian oxygen disaster" occurred much earlier [a good two billion years ago], the first known great glaciation [variscium] was over half a billion years later). By contrast, the development of the various animal species took place within a few tens of millions of years, in the Cambrian period.

It appears to be much easier to vary internal organization within an already differentiated multicellular organism with the aid of the *hox* genes, which are practically universal in the animal kingdom and virtually identical even with regard to their sequence, than to achieve the mutual compatibility of different cells in the first place. Obligatory cooperation also entails risks, which are brought to light by SNA (cf. SNA investigation of coupled loops [hypercycles][15].

Apoptosis plays an important role in the suppression of tumour growth (some cytostatic agents act by selectively increasing the apoptosis of tumour cells of at least certain kinds; they include cis platinum {cis-$[PtCl_2(NH_3)_2]$}) and also in the development of the embryo and foetus, for example, in the formation of the fingers and the furrows of the brain.

[34] Expression of the genes and the formation of certain substances may also be induced by the occurrence of certain agents. This increases the efficiency of metabolism, since fewer (ideally no) proteins are produced that currently have no function.

[35] Only about 30% of enzymes are metalloproteins[26] in the sense that they have a metal ion at their centre that is decisive for their catalytic function. (This does not exclude the possibility that it can be replaced by certain others at least without total loss of function in some cases; there are in fact instances in which "exotic" ions such as Ni or the platinum metals, together with the apoenzymes, constitute more active catalysts than the native enzymes [ibid.]). But *every* substitution of this kind (e.g., of molybdenum replaced by wolfram) reduces the formal autocatalytic order of the original metal ion for the organism (even if it is chemically successful when regarded in isolation). It would seem that the evolutionary optimisation of enzymes in individual cases is only worthwhile if any central ions to be newly introduced already have other functions in the organism. There are scarcely any isolated functions of metals in an organism but apparently always whole sets of functions. This also applies to less common central ions of enzymes such as Mo, Co, and Ni. In terms of SNA, this is readily explained considering the requirement that the corresponding AC order brought about by and related to a certain metal or non-metal involved in catalysis (enzymes) or replication) be larger than the AC order of (at least non-sexual) reproduction, that is > 2 [21] to maintain the cycle in mortal organisms.

But in the light of SNA there are two aspects that constitute possible therapeutic approaches:

a) Strong cycles always show unstable, non-linear, sometimes oscillatory, and even chaotic behaviour. This chemical-kinetic chaos[36] has long been known to exist in metabolic processes, too.
b) Metabolism in tumours is often greatly simplified; in spite of the tumour cells' ability to divide, individual metals have far fewer functions within a tumour than in the complete metabolism of the organism (since the tumour no longer fulfils certain functions of the organ).[37]

So the basic equations of autocatalysis apply to a tumour in particular, *without restriction* by control factors such as those designated Y[38] or W[39] in the Eiswirth classification. A tumour should therefore be unusually "vital" (as long is it does not die together with its host); this is reflected in its proliferation, which may extend into some differentiated tissue of another kind (metastasis). Only during *therapy* are the control species Y and W introduced on the nucleic acid or metabolic supply (iron or transferrin antagonist) level. This is one of the typical additions of part-systems in SNA; the cycle influenced by the cytostatic agent may quite well remain critical provided that it is not bridged or "short-circuited."

1B becomes 1CW with a change in the phase relationships between some biochemical signal fed into the autocatalytic loop and the corresponding answer, e.g., in terms of cell division under therapy; at the same time, the capacity for autonomous oscillations should be blocked. The AC cycles for certain chemical elements or species[40] are then *no longer strong/critical* (even without external influences such as an attack by the immune system or chemotherapy with a competitive approach) if they have only one or two functions left. The tumour (apparently nearly every tumour) also leaves a biochemical "trace" in the serum (both with regard to Cu and Zn levels in the serum and to the ratio of the two ions)[27].

Accordingly, when certain functions are fulfilled by two similar metals (e.g., Zn and Cd in algal carboanhydrases or Mo and W in redox enzymes of bacteria), the metalloproteins are grossly different from each other in both size and amino acid sequences, avoiding direct mutual replacement and possible violation of stability criteria for autocatalytic systems as inferred from SNA[21].

We may also assume that both the acute toxicity and the mutagenicity of metal ions are determined largely by interaction(s) with metal ions other than the physiological ones. This does not mean that the 70% metal-free enzymes or proteins can be disregarded in the long term. But they are not the subject of the present study.

[36] In such cases the kinetics of the reaction are neither classically linear nor exponentially kinetic nor explosive due to autocatalysis (branched radical chains [detonating gas] or similar) nor periodically oscillatory. Even if the experiment is repeated numerous times, the kinetic course of the reaction is never predictable or reproducible. The first reaction of this kind was found to take place in glycolysis with yeast cell extracts (around 1960). At that time chemical oscillations in solution systems (rather than at interfaces such as electrodes) were generally considered extremely unusual or even highly doubtful from the empirical point of view, but we now know of several "inorganic" systems with the same properties. Most of them are based on chlorine dioxide or $ClO_2^-$ as an oxidizing agent, e.g., with iodide or thiocyanate and malonic acid as a second reducing agent, and chemical chaos is well established even in gas phase systems.

[37] This does not exclude the possibility that hormone production will be irregular at the same time. (Numerous genital gland and pituitary tumours can be detected early in this way, especially in children and elderly patients.)

[38] If Y does not lead to regeneration but only to a withdrawal of catalyst, this results in Type 1 C (C standing for continuous flow) that has several subdivisions and only oscillates in a flow system corresponding to the biological case. Even in a "simple" autocatalytic system Y may be a number of different species $Y_1...Y_n$ which exert control collectively in the same or a similar manner, whereas in 1 CW W is always alone and thus clearly defined.

For non-trivial (e.g., acid catalysis of oxidation) systems such as the citric acid cycle, X is also a collective of intermediate products that together make up the "X" in the sense that it is possible to enter the autocatalytic cycle at any point, after which various merging forms of X act as $X_1 \ldots X_n$ with the other components entering.

[39] W in the cycle type 1CW constitutes a third loop capable of intercepting essential control and feedback substances (see Fig. 2).

[40] Phosphate as a nucleic acid skeleton, as ATP, as calcium phosphate in bones … must be considered separately. Each of these does contribute equally (by one) to the autocatalytic order of P which can be defined from the various functions as those entail different functions without being capable to replace each other in a given speciation form.

### APPLICATION TO BIOLOGICAL SYSTEMS: THE SIGNIFICANCE OF AUTOCATALYSIS (NUCLEIC ACID REPLICATION, CELL DIVISION, REPRODUCTION), STRONG AND CRITICAL CYCLES

In the case of coupling with other cell types (i.e., the combination of morphologically and functionally differentiated cells in one organism), the individual, potentially strongly autocatalytic cycles become a set of mutually dependent critical[41] cycles; the stability that existed when the products[42] (individual products) obtained from the individual cell types (autocatalytic cyclic systems) were identical and must be maintained by additional measures to prevent an undesirable, if not lethal, internal selection (i.e., the death of one type of cell/one organ) if three[43] or more types of cell are linked. Here it must be taken into account, and it is "useful," that couplings of autocatalytic units (cells) in a heterogeneous environment differ in some respects from the simpler consideration of hypercycles in a spatially homogeneous environment. Because the tissue, which is no longer differentiated, fails to perform metabolic functions, the tumour may be regarded as a parasite — one that "acts" in a cytologically, morphologically, and biochemically inhomogeneous environment. It has been shown for a parasite of this kind[28] that the removal of material and uncontrolled copying of nucleic acid in a spatially differentiated environment can be prevented by the formation of *chemical waves*. So the transition from cross-catalysis in multicellular organisms to mere autocatalysis in the tumour involves a risk for the parasite if the latter triggers chemical waves[44] by its divergent biochemical behaviour or under therapy. Because of their ability to oscillate even in "vessels" without a steady inflow of chemicals that indicate pronounced autocatalytic activities, most 1B systems are capable of forming such chemical waves; and as we have seen, the nucleic acid-polymerase system to be controlled is also of the 1B type[45]. In many cases cell division is also controlled by chemical waves (including $Ca^{2+}$ concentration waves that propagate on cell walls).

### THE SCOPE OF VALIDITY OF SNA IN BIOLOGY/MEDICINE

A further aspect is that stoichiometric network analysis is independent of individual hierarchies. It can therefore be applied in uniform biological and ecological terms from metalloproteins to biogeochemical cycles within and between whole biocoenoses[18] and enables causal substantiation of established biochemical and ecological rules of thumb, including those of population dynamics. But numerical approaches of this kind are of very limited use in a general

[41]The critical nature of the mutually dependent cycles derives from a simple dynamic condition for stability resulting from the fact that the (hyper)cycles/cell types exchange material among themselves. If the "numbers" of the two linked cycles are exchanged (they have no physico-chemical significance as such), it follows from the requirement of stable cooperation that the autocatalytic and exit orders in the two loops must all be identical to each other, i.e., each one individually and also the hypercycle formed from them is a critical system in the meaning of the SNA nomenclature (structural details are without significance for the formal classification[15]. This is also the reason for their susceptibility to disruption, which can be observed in symbionts beyond the organismic level (lichens, the zooxanthellae in coral polyps).

[42]For example, the two-coupled cycles doubtless produce carbon dioxide and water as identical end products of biochemical oxidation.

[43]It was assumed until recently that symbioses of more than two different types must in general be dynamically unstable. Not long ago an exception was discovered in a marine worm: in this case the balance between sulphate-reducing and $H_2S$-utilizing endosymbionts is achieved by exploiting a difference between the redox potentials of various organs of the worm.

The simplest multicellular organisms (certain coelenterates) have only two types of cell, apart from a few sensory and nerve cells. One of these types makes up the "inside" and specializes in digestion. By contrast, human beings have about 200 different cell forms. So in vertebrates, arthropods, etc., there is a regular logarithmic relationship with genetic complexity.

[44] Chemical waves also require autocatalysis, and they may be accompanied by oscillations, but the conditions for this are less strict as far as substances and concentrations are concerned.

[45]Chemical waves have already been observed in vitro in RNA replicase systems.

discussion of the problem of normal biochemistry, especially of uncontrolled cell division, because they assume a detailed knowledge of the kinetics of individual enzymatic reactions that is only available in such diversity for a few species, although a number of formalized approaches to the biochemistry of simple organisms such as *Saccharomyces cerevisiae* have been made in the Netherlands (e.g., [29]).

In our opinion the *theorem-based* "axiomatic" approach[14,15,30] in which the theorems are used directly through autocatalytic or cross-catalytic systems will be preferable to numerically based matrix calculations in nearly every case in the foreseeable future when it comes to handling biological systems, including pathological systems as in the problem of cancer and chemotherapy.[46]

Because a malignant tumour has escaped from regulation of general metabolism and in particular from regulation of its cell division by the rest of the organism, it (or, rather, its dynamic and autocatalytic features including the corresponding AC orders for different chemical elements) must be investigated separately, as an independent unit, by stoichiometric network analysis. It is an advantage also in terms of chemotherapy[47] that its metabolism is usually simplified. This facilitates even fundamental changes in the autocatalytic process and is thus the precondition for chemotherapy with metal salts and similar substances.

### CLASSIFICATION OF THE BASIC BIOCHEMICAL PROCESSES OF CELL DIVISION IN THE SNA NOMENCLATURE: CRITICAL CYCLES OF THE 1B TYPE

A distinction is made between various categories of substances (that are not essential for oscillation but may interfere with it) that can be made to intervene in a strong or critical autocatalytic system[20]:

a) Species whose concentration does not oscillate, which yet may produce substances necessary for the cyclic process and any oscillation but are otherwise only involved in secondary reactions
b) Species that may themselves oscillate but whose varying concentrations have no influence on the AC cycle and that are stable end products in borderline and extreme cases
c) Species that react with at least one important component or appear once as a reactant and once as a product
d) "Essential" species, the addition of which may cause phase shifts

Types a) to c) must be regarded as ideal variants that can scarcely be identified in real chemical systems.

[46]N.B.: This is a problem not of currently available or soon to be expected computer power or the complexity of biological systems, but of our imprecise knowledge of the kinetic data of individual biochemical reactions. The precarious situation of the tumour in the catchment area of chemical waves, with simultaneous changes in the (temperature-related) reaction kinetics, is shown by some successful attempts to fight tumours simply by raising the body temperature (hyperthermia treatment after von Ardenne, still at the experimental stage). In spite of excessive cell division a tumour represents an extreme metabolic condition, especially under conditions given by the very autocatalytic behaviour.

[47]This is one of the peculiarities of autocatalytic systems that the latter owe to their feedback loops: though a simplified metabolism seemingly provides less different way to interfere with, the tumour actually becomes more sensitive towards competition effects like those exerted by gallium or Ru(III) salts as there are more elements close to the lower threshold of stable, that is, strong or critical AC cycles than in the complete healthy organism where this holds only for Mo, V, Ni, and a few others.

### STABILISATION AND DESTABILISATION

Stabilisation of an autocatalytic cycle means that the system is shifted out of the range of oscillations into a condition in which oscillations no longer occur. Conversely, destabilisation is a process in which oscillations can be induced by means of additions outside the normal oscillatory parameter (concentration) range. When establishing the model the species X and Y are generally assigned empirically according to which stabilising components (nevertheless) result in unstable extreme currents[48] in the reduced diagram (method and examples in [20]); in general this is only possible with some of the possible permutations between X and Z (in this case, between the functions of the polymerases and nucleic acids).

### AUTONOMOUS OSCILLATIONS OF THE SYSTEM AND UNCONTROLLED CELL DIVISION

The question is whether chemically autonomous oscillations induced in the nucleic acid-polymerase system itself result in cell division which they become uncoupled from the control of the body as a whole and thus potentially cause tumour formation, or whether (conversely) they themselves are the initiator or one of the initiators of cell division. However, the latter is usually induced by very specific chemical signals from other cells, as in the healing of wounds, which means that the first assumption would seem more likely; moreover, differences in the concentration of certain reactants would otherwise lead to different oscillation frequencies or bistable behaviour, i.e., loss of biogenic rhythm in cell division.

The assumption that autonomous chemical oscillations in the NA-polymerase system are *harmful* will therefore be taken as *a working hypothesis* for the *effect of cytostatic agents* too. According to this, stabilisation would be tantamount to prevention of autonomous oscillations in concentration and those of the activity of single-stranded DNA; moreover, either direct or inverse regulation is possible. But critical cycles such as those we are considering here only behave in an unstable (e.g., oscillatory) manner in the presence of other destabilising influences (carcinogenic activity of chemicals[49]).

The basic features of critical autocatalytic cycles are visible in the context of carcinogenesis to the extent that besides the uptake of an element that is essential but has a carcinogenic effect in excessive amounts (Se, Ni) or in the “wrong” level of oxidation (V, Cr[50]) or in the “wrong form” of speciation (e.g., Co if not bound to corrin rings), the reabsorption of this element — for example, as a result of complex formation — often plays a role in homoeostasis, too. In this case the cycle species (the intact metalloprotein) *X* is first transformed into a subsequent product *Z*, which can then be made usable to the cyclic process again (at least partially) through reaction with a third species *Y*.

[48] “Extreme currents”[22] are the “leanest” flow and interaction diagrams, starting from a real cycle, that still show the full dynamics (e.g., oscillations) of the complete diagram or reaction mechanism. SNA also serves to identify such extreme currents with and in complex reaction mechanisms or other causal networks (such as the global climate system[17] to make such systems capable of description.

The method used *ensures* [16,30] that no over-reduction takes place in the sense that any aspects of the dynamics possible to the modelled system are lost. This is essential here in the light of the working hypothesis that autonomously induced oscillations are involved in the genesis of tumours. A model in which such oscillations no long occur (can occur) would not be an adequate description either of carcinogenesis or of the intervention of possible cytostatic agents. We therefore insist that the use of SNA and its theorems is one of the best methods of depicting these dynamics reliably (cf.[16]).

[49]Ultimately, the carcinogenic effect of ionising radiation is also due to chemical reactions [production of free radicals or reactive ions].

[50] In this case the oxoanions of the highest level of oxidation that are very easily transported and utilized biologically in addition to or instead of phosphate or sulphate. Empirically, there is also a relationship between the oxidizing effect (redox potential) of (various) metal ions and their acute toxicity[31].

A configuration of this kind corresponds to Type 1B, which also permits oscillations[51] in a half-closed system (B = *batch*).

An example of such a Y species is the methyl group (bound to cobalt in vitamin $B_{12}$), which irreversibly converts selenium (whether inorganic [e.g., as $SeO_3^{2-}$, its most toxic form] or as selenoamino acid) into the trimethylselenonium ion $(CH_3)_3Se^+$, which is excreted in the urine. Such systems also require a constant supply of precursors of autocatalytic-loop species (selenium compounds in this example); besides the actual reactants the flow system requires vitamins, so to speak. Here we have Type 1 CX[52]. It is always present when the above constant supply is necessary for oscillatory behaviour (which is relatively difficult to investigate in biochemical systems; cf. [22]).

The activity of a system of nucleic acids and DNA polymerase can be classified as Type 1B because an inactive form initially emerges from/with the matrix (through pairing[53] with the negative strand), which is only returned to the cycle after interaction with Y (the polymerase/transcriptase, etc.).

For the *systematic design of a chemotherapy*, hence it is necessary to seek possible modes of interference in a 1B system of this kind as well as suitable binding partners for nucleotide bases among the metal complexes. Cytostatic agents may also break up the coupling between the two part-loops if the nucleic acid modified by metal addition is no longer recognized as a substrate by polymerases or no longer accepted by the polymerase or other ribosome components for stereochemical reasons. So in the 1B cycle the species designated as Z must be regarded as an intermediate product of the outer part-loop during the partial transformation of X into Y, which drives the "real" autocatalytic cyclic process and controls it by branching the position of Y.

[51]In our context it is not only or primarily a question of oscillations as in glycolysis, which are also thought to be connected with the "internal clock."

Cell division, especially the replication of genetic material, involves the periodic activation of certain enzymes (e.g., nucleic acid polymerases), which may be regarded as oscillatory behaviour (the periodic increase in the concentration of certain components such as free nucleic acids). Because only the concentrations of intermediate products ($H_3O^+$, elements in certain levels of oxidation [often iodine oscillating between the elemental or triiodide and the iodate state]) and those of companion substances (e.g., complexes with indicators) can oscillate, not those of the educt or product (i.e., no oscillations whatsoever around the chemical equilibrium are possible [Onsager principle]), the maximum concentrations of intermediate products such as messenger RNAs are especially significant. Moreover, several essential elements suppress cell division and reproduction if overdosed (molybdenum, zinc; in plants also phosphorus[32]).

[52]The classic clock reactions ("chemical clocks") also belong to this category, with or without a modification that may result in oscillatory behaviour[20].

[53] Only the single-stranded form is active in the sense that it replicates or can be used for the purpose of controlling protein synthesis; the double-stranded form (whether it is present as a helix) also contains the genetic information, but it is not the autocatalyst. (In this case the term "autocatalyst" is restricted to the kinetically/chemically active species; it does not include the whole range of stable intermediate stages from which the autocatalyst can be released by redox or other reactions [in reactions with an acid autocatalyst, for example, only the proton or oxonium ion $H_3O^+$ is regarded as an autocatalyst, not water from which it is usually formed]).

Not until the reaction with the polymerase or transcriptase does it return to the AC cycle where it has a *productive* effect; it does not break down the autocatalyst, for example. So here we have the cycle type *1B* again, not one of the 1C variants (i.e., neither 1CX nor 1CW nor 1CS [special case for more complex oscillators such as the Roelofs-Jensen system]). The Roelofs-Jensen oscillator, consisting of benzaldehyde, atmospheric oxygen, $Co^{2+}$, bromide ions, and acetate as a base in which the concentrations of all cobalt(III), benzoyl radicals, and dissolved oxygen oscillate[33]) is not one of the "simple" systems[20]; its (cobalt-based) core cycle is strong. However, it does have two coupled autocatalytic cyclic processes.

In the case of an "addition" (combination of the components) of two (in Eiswirth's sense of the term) simple oscillators (e.g., of iodine/iodate/$H_2O_2$[34]) with the malonic acid/Mn(II)/iodate system to form the Briggs-Rauscher oscillator) this (the transition to 1CS or similarly complex patterns) does not necessarily occur (for the Briggs-Rauscher oscillator is also of the 1B type). This is a further strong indication that the method can be applied to the far more complex biochemical systems formally 1B + 1B.

Similarly, individual processes can best be isolated and described separately as in this case (Type 1B) critical cycles if they are based on sub-units that process information in the same manner and are chemically fairly selective. In this case it is the nucleic acids and ribosomal proteins that assume such a role.

### PRACTICAL APPLICATIONS

If we regard the replication of polyphosphoric acids functionalized in a particular manner, namely the nucleic acids, as the (assisted) autocatalytic process in biology which is central to our investigation (as not only the fundamental significance of genetic information but also the sites of action of the cytostatic agents and numerous carcinogenic agents discussed here suggest), the effects of substances that:

a) break down the polyphosphate chain directly (Be, As, V(V)),
b) attack the histone proteins enveloping or protecting the DNA, or
c) inhibit nucleic acid synthases (such as DNA polymerase) {case $c_1$), e.g. $Ni^{2+}$} or cause over-activity with consequent inaccurate copying of the replication {case $c_2$); Cr(III) formed from chromate in the cell nucleus must be examined}.

These effects can be described directly on the basis of the Eiswirth nomenclature once again.

Beryllium salts attack polyphosphate chains directly[35] (like certain compounds of arsenic and presumably of vanadium by including the pentavalent ions into polyphosphate ions where they then undergo reduction cleaving the chain eventually) and have a direct mutagenic effect by splitting the nucleic acid chains. Moreover, carcinogens can influence the role of enzymes in the autocatalytic process, which may be either accelerated or inhibited. Additions to DNA can change the cyclic nature of the replication of nucleic acids simply because the coordinative binding to nucleotide bases and the blocking of H-bridge acceptor positions leads to an incorrect pairing of bases. A formally simple[54] image/mirror-image process with negative matrices in the synthesis of nucleic acids is thus changed in such a way that a different basic (reaction-topological) pattern and a correspondingly different dynamic pattern result even after the complexity has been reduced by SNA, which in this case leads to type 1B.

Case a) in the above list corresponds to a direct exit from the autocatalytic X cycle (arrows pointing left or top right in the complete diagram [not that of the extreme current, i.e., the left one of the pairs of equivalent diagrams]); perturbations according to c) act on or through Z. A decrease in the accuracy of copying from cases b) and $c_2$) corresponds to increased direct exit (outflow of the autocatalyst by producing NAs no longer useful for replication); this tends to weaken the cycle. But X has positive regulation (see below). Increasing exit to “top right” direction (by combining with Y) affects interaction with the polymerase and thus the extent of negative feedback (which depends on the occupation of the enzyme by the substrate); because of this and the positive regulation the replication process may become oscillatory and unstable and thus possibly contribute to tumour formation.

The result of an intoxication that directly attacks nucleic acids at their polyphosphate backbone (e.g., by $Be^{2+}$) or renders them useless for replication (by hydroxylation or de-amination of nucleotide bases [nitrite, OH-radical-forming metal carcinogens like Co, Ni, or Pb]) turns the 1B pattern into one of 1CX type. What then happens is obvious from an inspection of table 4 (see below): whereas the kind and conditions of possible destabilising effects on X remain unchanged, this is different with Z, that is, with some or any species required to maintain the autocatalytic cycle by matter supply, including nucleoside triphosphates: decreased supply of these now tends to stabilise rather than destabilise the X cycle.

Accordingly the system will run out of control now once there is enhanced metabolic activity, e.g., in response to stress. In such a 1CX system, e.g., $Be^{2+}$ will act as a pathological (and

[54] Here we are considering the normal case of multi-stage replication of any arbitrary sequences, not the special case of the direct depiction of palindromic RNA, i.e., of sequences that are identical to their negative apart from the ends. But palindromic part-sequences are typical sites for the action or recognition of RNA replicases and also play a role in cell-division control and termination by telomers.

pathogenic) Y bringing about secondary species that in turn decrease efficiency of NA replication. Unlike the physiological case, such compounds do not introduce a Z (or $Z_{1B}$, respectively) in due control of the cycle (do not confuse the different roles of the Z species in 1B and 1C cycles). It remains to be investigated whether there is a clear-cut "switching" between the "healthy" 1B and pathological 1C state or cycle topologies and how it is influenced by metabolic stress including NTP levels.

Allocation to different paths corresponds to different *phase relations*[55] between "interference" and the autocatalytic cyclic process; in the case of chemotherapy and physiological regulation (e.g., apoptosis) a specific intervention by antagonists of certain essential metal ions or complex ligands may constitute such interference. If we know the principle by which a toxin or cytostatic agent attacking the nucleic acid works, for example, by cross-bridging in the case of cis platinum, we can draw conclusions about certain phase relations between the interference and onset or removal of non-linear chemical behaviour, possibly even with regard to the phases of cell division in which the substance is especially effective or dangerous.

This suggests a novel approach: modifying the effect of cytostatic agents by intervening in the transport processes[56] that carry the active substance (generally speaking) into the cell nucleus before or during division. It is probable that the carcinogenicity of nickel compounds[10,37] that do not dissolve readily and of chromate(VI) is connected precisely with triggering of such phase effects rather than the presence of excessive amounts of Ni or Cr in the cell nucleus.

### CLASSIFICATION OF METALLOID AND METALLIC CARCINOGENS

The following metal (metalloid) ions are known to have a carcinogenic effect on humans (among others, and in addition to the actinoides such as plutonium, which are also radiotoxic). The sites of the tumours are shown in brackets.

Other substances under discussion as possible carcinogens are the highly toxic[38] $In^{3+}$, rhodium salts[35], and some other elements. V, Cr, and Ni are also thought or known to be essential to man, although on other levels of oxidation in some cases; Cd is essential for other mammals (goats[39]). The effects of overdoses may be responsible for carcinogenesis here.

[55]In the chemistry of oscillators it is usual to proceed in the opposite direction to explain the reaction mechanism: agents are added that may "interfere" with the oscillations (e.g., side products or end products [$Br^-$, $O_2$, formate, certain organic halogen compounds such as halogenated malonates]) and whose removal — e.g., into a supernatant gas phase or into the outlet in a flow system — is empirically necessary for maintaining oscillations in the system under scrutiny. From this the nature and possibly the extent of phase shifts in chemical oscillations or the limits of concentration and temperature at which these break down completely are determined. For this point the conditions of chemical kinetics can be stated precisely([36]; bifurcation criterion).

In particular, work of this kind has been carried out with the numerous variants of the Belousov-Zhabotinsky oscillator and has led to further studies of a similar nature. As a rule the BZ variants use malonic or citric acid as a substrate, bromate as an oxidizing agent together with acid and a redox-active metal ion [as a co-catalyst] to which other CH-acid compounds (e.g. cyanoacetic acid, acetone, barbituric acid or malic acid) or further reducing agents (oxalic acid, iodide) or bromide-precipitating agents ($Ag^+$, $Tl^+$, $Pb^{2+}$; these stimulate oscillations) have been added as influencing reagents.

As described above and on the basis of such studies, Eiswirth et al.[20,22] classified several dozen systems known to be chemically oscillatory and quite a number of model mechanisms which should theoretically cause oscillatory behaviour. The above findings strongly suggest that the classification method and the concept of interference can be applied directly to mechanisms of carcinogenesis and chemotherapy.

[56] Here we are thinking in terms of selective coupling to receptors of transport proteins, preferably via anchor groups that are small enough to "fit" into a receptor, or uptake by means of phagocytosis.

**TABLE 3**
**Inorganic Carcinogens and Their Mode and Site of Action**

| Ion/Element | Site of Attack/Site of Tumour |
|---|---|
| $Be^{2+}$ | Lung, bones; inhibits DNA polymerase |
| $Cd^{2+}$ | Kidneys |
| Nickel, esp. in hardly soluble forms | Lung, nasal mucosa |
| Chromate(VI) | Respiratory tract[57], skin |
| Arsenic | Mouth, larynx, oesophagus, bladder |
| Selenium | Liver |

### TYPE 1B CYCLES, "SHORTCUTS" IN THE AC CYCLE AND DETOXIFICATION: IN WHICH CIRCUMSTANCES CAN AN ESSENTIAL ELEMENT CAUSE CANCER?

Although it is probably correct to assume that the cycle type 1B is found in most concentrations of any chemical element in biology, the shape of the practically universal dose-effect curves does imply that detoxification mechanisms with metallothioneines, glutathione, and possibly biomethylation (change of speciation) either take on the function of additional sinks Y or at least modify the formally critical character of the cycle without its becoming weak[58]; this would result in the decline of the pre-tumour because of a lack of cell division (and maybe that is the case as a rule). We may conclude from this that if the detoxification processes fail; they do not result in a "bridging" or "short-circuiting" of the usual critical 1B cycles. So the (reversible) methylation of selenium or arsenic (both essential to man) does not break up the central AC cycle with X or shorten it by removing certain parts of the process.

The manner in which carcinogens may interact with nucleic acids as autocatalytic units, for example, through intercalation in the helices, or coordinative binding to bases, is important both for the induction of neoplasms/tumours and for chemotherapy (e.g., with cis platinum or Budotitan® derivatives[59]. An acceleration of polymerase activity, e.g., by chromium(III), may lead directly to excessive cell division but also to a loss of precision in nucleic acid copying.

### CLASSIFICATION OF CHEMOTHERAPEUTIC AGENTS

According to this, the objective of chemotherapy would be to force a transition from an autonomously oscillatory regime to a stable or bi-stable regime, after which the tumour would stop growing and be controlled by immune reactions. Conversely, carcinogenic agents should have a destabilising effect on the cycle. The following table applies to critical cycles[22]:

Direct regulation takes place if the effect increases with concentration; otherwise, regulation is indirect. Assuming that the cycle type is 1B, it is now possible to examine whether the addition of certain species such as the said cytostatic agents has a "stabilising" effect on the two-loop autocatalytic cyclic process in that cell division is no longer possible or at least requires

[57] After inhalation of dusts of chromates or of $CrO_3$ or that of $CrO_2Cl_2$ vapour.

[58] In fact, either is possible: destruction of the central AC cycle by making it weak, suppression of endogenous oscillations or transformation into 1CX states. But practically the second kind that maintains the 1B cycle will prevail because (relatively) low doses will provide this result and there is a limit in dosing because of the usually severe side effects of chemotherapeutic agents.

[59] bis-benzoylacetonato-bis-ethoxytitanium(IV)); inorganic Ti, into which this may be converted in vivo is extremely effective as a proteinase or peptidase inhibitor — a property which can be exploited therapeutically to combat bacteria, viruses (including HIV), and also tumours[11].

additional chemical signals from outside (even essential chemical elements such as iron can act as co-carcinogens). Y species with d+ behaviour are therefore probably both genotoxic and carcinogenic. An example would be Cr(III) formed in situ through reduction of chromate entering the cell nucleus, which would accelerate cell division.

Substances that reduce the activity of the nucleic acids, i.e., that act on X, have a stabilising effect of this kind that increases with concentration; so this corresponds to one of the above categories of cytostatic agents.

If Y is the single-stranded, active form of nucleic acid, further branching between a return to the cycle of X (in the double-stranded form) and loss, and thus (in this case undesirable) dynamic instability of the cycle including the ability to oscillate, depends on whether a modification has occurred. The s+ character of X (i.e., the double-stranded nucleic acid) that exists in all critical cycle types has the effect that any intervention that directly reduces the potentially active concentration[60] leads to destabilisation (and this results in genotoxicity). Consequently, it is possible to make a clear formal distinction between mutagenicity and carcinogenicity by means of SNA, although they often coincide empirically.

Platinum (cis platinum and its analogues and amine-iodoplatinum(IV) complexes liable to photochemical or glutathion-related reductions), titanium (inorganic) and rhodium(I) act on the first of the double loops (i.e., the "inner" one containing the autocatalyst X), whereas the metallocenes act on the second. Gallium and ruthenium compounds block the iron metabolism of the tumour, which means that they work on a totally different level of the system, leaving nucleic acids alone. But of course it is possible to analyze this functional level by means of SNA too. It is not yet known by which mechanisms organic germanium and tin compounds act against cancer cells.

### ATTACK ON DNA

During their coordination or adsorption to the nucleic acid[61], most metal ions (not only the genotoxic ones) change the direction of the latter's "spiral," i.e., the helicity of certain DNA sections [10]; this effect stands in a qualitative relationship with the coordinative affinity for O on the one hand or N, S on the other (cf. the thiocyanate model[21] that links acute toxicities of

[60]This simplified formal description implies that the exactness of replication in a disturbed system remains sufficiently high (below the critical size of the genome [given a certain mutation rate] [40]). If this is no longer the case, the result will be an abrupt fall in the active concentration and the system will start to oscillate under the effect of the increasing frequency of replication errors. These are mutagenic substances that, like chromate, also have a carcinogenic effect and activate polymerase. So what we have here is a kind of short-circuit between the two loops. That a short-circuit of this kind is not disastrous in every case is due to the fact that it usually weakens autocatalytic cycles at the same time. But the formal autocatalytic order of metal ions in polymerases is very high because these (Ca, Zn) fulfil very many functions in biology.

[61] In theory, at least, nucleic acids are mostly "*ambidentate*" ligands, because the metal ions can bind either to the polyphosphate chain (i.e., oxygen donors, to which especially lanthanoids, calcium [cf. the former use of polyphosphate complexes of $Ca^{2+}$ in water-softeners for washing machines] or aluminium are firmly bound) or (on the other side of the bridging sugar [ribose or deoxyribose]) to nitrogen atoms of the heterocycles (i.e. to the real nucleotide bases adenine, guanine, cytosine and thymine or uracil). To this extent there are parallels with the coordination of transitional metal ions such as Co(III) or platinum metals to other similarly ambidentate ligands such as urea, 4-hydroxypyridine, or formamide. In this case (with the NAs), linkage isomerism manifests itself as bimodality of the bond curve (strong binding to one of the donor centre groups even at low concentrations; subsequently, at higher concentrations, weaker binding to the other group also); specific binding preferences result in a simple sigmoid saturation curve.

A continuous transition from one bond direction to the other that gives rise to kinetically more or less stable linkage isomers (cf.[41]) is also found with other ligands such as nitrite or thiocyanate $SCN^-$ (also called rhodanide); the latter can be correlated with quantitative properties of the ions[42]. In this case there is a qualitative relationship between the acute toxicity of an ion when administered orally to rodents and the direction of the bond between $SCN^-$ and that metal ion[21]; this indicates that a preferred coordination to N can *prevent* an attack on sulphur-containing functional groups such as disulphide bridges, the effects of which would be much more damaging not least because such an attack is presumably almost irreversible[43] .

**TABLE 4**
**Autocatalysis, Regulation, and Stability in Critical Cycles**

| TYPE | X | Y | Z | W |
|---|---|---|---|---|
| 1B | s+ | d+ | d- | |
| 1CW | s+ | d+ | s- | s+ |
| 1CX | s+ | d+ | s- | |

metal ions and organometal species to the binding directions towards $SCN^-$ ions), but not with carcinogenesis.

As far as changing helicity[62] is concerned, Ag, which has a pronounced affinity for both sulphur and nitrogen, is more effective than the oxophilic $Al^{3+}$, which binds to the "outside" (that is, within the cell water-exposed) polyphosphate chain. In the case of nickel it must be taken into account that the uptake of species that are not readily soluble, such as the sulphides, or of NiO occurs by phagocytosis that leads to high intracellular concentrations and correspondingly massive reactions in the sense of tumour induction, whereas soluble nickel salts are also taken up but have only a slight carcinogenic effect or none at all. Presumably the resulting Ni concentrations in the cell nucleus are lower in this case than when more or less compact particles are taken up.

### ATTACK ON POLYMERASES

In a 1B system there is a further possibility of regulation via some agent Z: destabilisation occurring at falling concentrations, i.e., by means of antagonists for the polymerase as agents that destabilise the cycle.

The apparently paradoxical result is that even substances that inhibit DNA polymerase (may) have a carcinogenic effect; examples are nickel and beryllium salts. At the same time this substantiates their allocation to Type 1B; a further argument is that tumour cells can be cultivated in largely closed systems.

### OTHERS

In some cases chemotherapy may also simply mean "tuning down" marginally strong cycles whose marginally strong status is due to the fact that the tumour cells usually have a simplified metabolism. An example is the inhibition of iron utilisation within the tumour by gallium salts, a condition for this being the autocatalytic order in respect of Fe and thus the number of enzyme functions related to iron within the tumour low enough[63]. This is also a result of the reduced complexity of the tumour's metabolism as compared to that of the organism as a whole, in

[62]Yet there is no direct relationship between change of helicity ("re-spiralling") and carcinogenesis or cytostatic behaviour although conceivably the action of topoisomerases will be influenced by this kind of metal ion activity. It is rather related to acute toxicity[10].

[63]An analogy in healthy organisms is antagonists of those metal ions whose *number* of physiological functions (which has nothing to do with the significance of the individual functions) and thus whose autocatalytic order is low in any case, such as molybdenum and cobalt that can be inhibited by wolfram and arsenic.

conjunction with a loss of regulation by other types of cell. At the same time this necessitates a "separate" SNA analysis of metabolism in the tumour.

### CONCLUSION

SNA provides an aid to understanding key processes and phenomena of carcinogenesis and the chemical treatment of tumours. The processes that control cell proliferation can be allocated to a certain type of critical cycle, namely 1B. The phase relations for intervention reveal prospects for more specific treatment of tumours. The diversity of the metal-containing chemotherapeutic agents can be reduced systematically to just a few of the modes of action which are conceivable from the point of view of SNA. The basic type of biological autocatalytic loop has been identified, and this permits new designs of chemotherapeutic agents. Moreover, we expect in the future to define novel strategies for finding such chemotherapeutic compounds.

## References

1. Zander, M. (1995). *Polycyclische Aromaten — Kohlenwasserstoffe und Fullerene*. Stuttgart, Germany: Teubner.
2. Gabel, D. (1994). Present status and perspectives of boron neutron capture therapy. *Radiother. Oncol.* **30**, 199–205.
3. Haritz, D., Gabel, D., and Huiskamp, R. (1994). Clinical phase-I study of $Na_2B_{12}H_{11}SH$ (BSH) in patients with malignant glioma as precondition for boron neutron capture therapy (BNCT). *Int. J. Radiat. Oncol. Biol. Phys.* **28**, 1175–1181.
4. Fränzle, S. and Grossman. (1999). Aufbau von Erfolgskonfigurationen in Wirtschaft und Umwelt mit kreuzkatalytischen Netzen. In *Nachhaltigkeit – Bilanz und Ausblick*. Grossman, W.D. Eisenberg, W., Meiβ, K.M., Multhaup, T. Eds.Frankfurt/Main, Berlin, Brussles, Bern, New York, Vienna, Lang, pp. 147–189.
5. Kopf-Maier, P. (1994). Complexes of metals other than platinum as anti-tumour agents. *Eur. J. Clin. Pharmacol.* **47**, 1–16.
6. Guo, Z. and Sadler, P.J. (1999). Medicinal inorganic chemistry. *Adv. Inorg. Chem.* **49**, 183–306.
7. Markert, B. (1994). The Biological System of the Elements (BSE) for terrestrial plants (glycophytes). *Sci. Total Environ.* **155**, 221–228.
8. Markert, B. (1998). *Das Biologische System der Elemente. Enzyklopädie Naturwissenschaft und Technik.* Landsberg, Germany: Ecomed.
9. Fränzle, S. and Markert, B. (2000). The Biological System of the Elements. Part II: a theoretical model for establishing the essentiality of chemical elements. The application of Stoichiometric Network Analysis to the Biological System of the Elements. *Sci. Total Environ.* **249**, 223–241.
10. Nieboer, E., Maxwell, R.I., Rossetto, F.E., Stafford, A.R., and Stetsko, P.I. (1986). Concepts in nickel carcinogenesis. In *Frontiers in Bioinorganic Chemistry*. Xavier, A.V., Ed. Weinheim and Deerfield Beach: VCH, pp. 142–151.
11. Schwietert, C.W. and McCue, J.P. (1999). Coordination compounds in medicinal chemistry. *Coordination Chemistry Reviews* **184**, 67–89.
12. Boutwell, R.K., Takigawa, M., Verma A.K., and Ashendel, C.L. (1984). Observations on mechanism of skin tumor promotion by phorbol esters cellular interactions by environmental tumor promoters. In Proceedings of the International Symposia of the Princess Takamatsu Cancer Research Fund **14**. Zeist (NL), VSP.
13. Weinstein, J.N., Myers, T.G., O´Connor, P.M., Friend, S.H., Fornace, A.G., Kohn, K.W., Fojo, T., Bates, S.E., Rubinstein, L.V., Anderson, N.L., Buolamwini, J.K., van Osdol, W.W., Monks, A.P., Scudiero, D.A., Sausville, E.A., Zaharevitz, D.W., Bunow, B., Viswanadhan, V.N., Johnson, G.S., Wittes, R.E., and Paull, K.D. (1997). An information-intensive approach to the molecular pharmacology of cancer. *Science* **275**, 343–349.
14. Clarke, B.L. (1974). Stability of topologically similar chemical networks. *J. Chem. Phys.* **62**, 3726–3738.
15. Clarke, B.L. (1980). Stability of complex reaction networks. *Adv. Chem. Phys.* **43**, 1–215.
16. Clarke, B.L. (1992). Stoichiometric Network Analysis of the oxalate-persulfate-silver oscillator, *J. Chem. Phys.* **97**, 2459–2472.
17. Clarke, B.L. (1995). www.chem.ualberta.ca/faculty/clarke/htm.
18. Fränzle, S. (2000). Stöchiometrische Netzwerk-Modelle in der ökologischen Risiko-Analyse. In *Der ökologische Risiko-Begriff.* Breckling, B. and Müller, F. (Hrg.). Frankfurt/Main, Berlin, Wien: Lang, S. 161–177.

19. Basza, G. and Beck, M.T. (1972). Autocatalysis, cross-catalysis, self-inhibition and crosswise inhibition: Pathways into exotic chemical kinetics. *Acta Chimica Hungarica* **73**, 26–37.
20. Eiswirth, M., Freund, A., and Ross, J. (1991). Mechanistic classification of chemical oscillators and the role of species. *Adv. Chem. Phys.* **80**, 127–198.
21. Fränzle, S. and Markert, B. (2002). The Biological System of the Elements (BSE) — A brief introduction into historical and applied aspects with special reference on “ecotoxicological identity cards” for different elements (for example, As and Sn). *Environ. Poll.* 720, 27-45.
22. Eiswirth, M., Freund, A., and Ross, J. (1991). Operational procedure toward the classification of chemical oscillators. *J. Phys. Chem.* **95**, 1295–1299.
23. Ottley, C.J., Davison, W., and Edmunds, W.M. (1997). Chemical catalysis of nitrate reduction by iron(II). *Geochimica et Cosmochimica Acta* **61**, 1819–1828.
24. Peusner, L. (1983). Electrical network representation of n-dimensional chemical manifolds. In *Chemical Applications of Topology and Graph Theory*. King, R.B., Ed. Amsterdam, Netherlands: Elsevier.
25. Winfree, A.T. (1974). Rotating chemical reactions. *Sci. Am.* **230** (6): 82–95.
26. Höhne, W.E.. (1980). Metallionen in Struktur und Funktion von Metallenzymen. *Z. Chemie* **20**, 1-12.
27. Zhang, Z., Zhuo, H., Liu, S., and Harrington, P. de B. (2001). Classification of cancer patients based on elemental contents of serums using bidirectional associative memory networks. *Analytica Chimica Acta* **436**, 281–291.
28. Boerlijst, M.C. and Hogeweg, P. (1991). Spiral wave structure in pre-biotic evolution. *Physica D* **48**, 17–28.
29. Heijde, P. (1996). Systematic analysis of stoichiometric models including kinetics and metabolic control for yeast glycolysis and branches. www.bt.tudelft.nl/R_Proj/bpk/P_Heijde.htm
30. Clarke, B.L. (1975). Some theorems on chemical network stability. *J. Chem. Phys*. **62**, 3726-3738.
31. Lewis, D.F.V., Dobrota, M., Taylor, M.G., and Parke, D.V. (1999). Metal toxicity in two rodent species and redox potential: Evaluation of quantitative structure-activity relationships. *Environmental Toxicology and Chemistry* **18**, 2199–2204.
32. Fiedler, H.J. and Rösler, H.J. (1993). *Spurenelemente in der Umwelt*. Jena and Stuttgart, Germany: Fischer.
33. Roelofs, M.G. and Jensen, J.H. (1987). EPR oscillations during oxidation of benzaldehyde. *J. Phys. Chem.* **91**, 3380–3382.
34. Bray, W.C. (1921). A periodic reaction in homogeneous solution and its relation to catalysis. *J. Am. Chem. Soc.* **43**, 1262–1267.
35. Dulka, J.J. and Risby, T.H. (1976). Ultratrace metals in some environmental and biological systems. *Anal. Chem.* **48**, 640–653.
36. Schneider, F.W. and Münster, A.F. (1996). *Nichtlineare Dynamik in der Chemie*. Heidelberg, Berlin, Oxford: Spektrum.
37. Huang, X., Zhuang, Z., Frenkel, K., Klein, C.B., and Costa, M. (1994). The role of nickel and nickel-mediated reactive oxygen species in the molecular mechanisms of metal toxicity and carcinogenicity. *Environ. Health Perspect.* **102**, 106–113.
38. Bienvenu, P., Nofre, C., and Cier, A. (1963). Toxicite generale comparee des ions metalliques. Relation avec la clasification periodique. *Comptes Rendus* **256**, 1043–1044.
39. Anke, M., Arnhold, W., Langer, M., and Krause, U. (1990). The influence of the ultra trace element deficiency (Mo, Ni, Cd, As, V) on growth, reproduction performance and life expectancy. In *Trace Elements in Clinical Medicine*. Tomita, H., Ed. Tokyo, Berlin: Springer.
40. Eigen, M., Schuster, P. (1978). The realistic hypercycle. Naturwiss. **65**, 1–41
41. Burmeister, J.L. (1968). Linkage isomerism in metal complexes. *Coordination Chemistry Reviews* **3**, 225–245.
42. Fränzle, S. (1992). Photochemischer Ligandenaustausch auf Halbleiterkolloiden und in Ionenpaaren zur Carbonylierung und Synthese neuer CO-haltiger bindungsisomerer Thiocyanatohalogenokomplexe von Os(II), Os(III), Ir(III) und Mo(III) als Beitrag zum Verständnis bindungsisomeren Verhaltens [Thesis]. Christian-Albrechts-Universitαt Kiel, Germany.
43. Shaw, W.H.R. (1961). Cation toxicity and the stability of transition-metal complexes. *Nature* **192**, 754–755.

## FURTHER READING

Clarke, B.L. (1992 b): General Method for Simplifying Chemical Networks while Preserving Overall Stoichiometry in Reduced Mechanisms. Journal of Chemical Physics **97**, 4066 – 4071

Fränzle, S.; Markert, B. (2000 b): Das Biologische System der Elemente: eine modelltheoretische Betrachtung zur Essentialität von chemischen Elementen. Die Anwendung der Stöchiometrischen Netzwerkanalyse auf das Biologische System der Elemente. UWSF – Z.Umweltchem.Ökotox. **12**, 97 – 103

Kawanishi, S.; Hiraku, Y. (1999): Metal Carcinogenicity: $H_2O_2$ production and DNA damage by δ–aminolevulinic acid in relation to lead carcinogenicity. www.3iwe.riken.go.jp/CONGRESS/SYMPO/SAT0120/AK0111/TIT.htm

Küng, A.; Strickmann, D.B.; Galanski, M.; Keppler, B.K. (2001): Comparison of the binding behavior of oxaliplatin, cisplatin and analogues to 5´-guanosin in presence of sulfur-containing molecules by means of capillary electrophoresis and mass spectrometry. Journal of inorganic Biochemistry **86**, 491 – 98

Zhang, B., Egli, D., Georgiev, O., and Schaffner, W. (2001): The Drosophila homolog of mammalian zinc finger factor MTF-1 activates transcription in response to heavy metals. Molecular and Cellular Biology **21**, 4505 – 4514.
